# Supplementary material for: Preparation of Large-Size, Superparamagnetic, and Highly Magnetic Fe3O4@PDA Core–Shell Submicrosphere-Supported Nano-Palladium Catalyst and Its Application to Aldehyde Preparation through Oxidative Dehydrogenation of Benzyl Alcohols
Source: Molecules. 2019 May 3;24(9):1730. doi: 10.3390/molecules24091730 (PMC6539375; doi:10.3390/molecules24091730)
Supplement: Supplementary file 1 [file molecules-24-01730-s001.pdf]

Supplementary Materials

Preparation of Large-Size, Superparamagnetic, and Highly Magnetic Fe_3O_4@PDA Core–Shell Submicrosphere-Supported Nano-palladium Catalyst and Its Application to Aldehyde Preparation through Oxidative Dehydrogenation of Benzyl Alcohols

Haichang Guo, Renhua Zheng, Huajiang Jiang, Zhenyuan Xu* and Aibao Xia*

Table of Contents

	Table of Contents	Page
1	XRD patterns, SEM images, and EDS spectra	2
2	Characterization of all products	3~4
3	NMR spectra	5~18
4	References	19

1. XRD patterns, SEM images, and EDS spectra

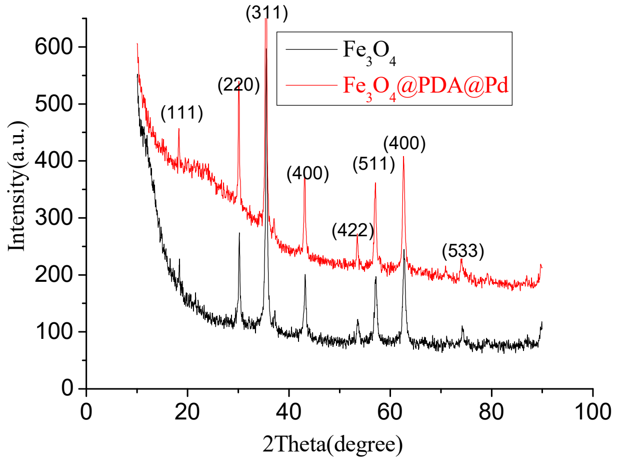

Figure S1: XRD patterns of Fe_3O_4 and Fe_3O_4@PDA@Pd

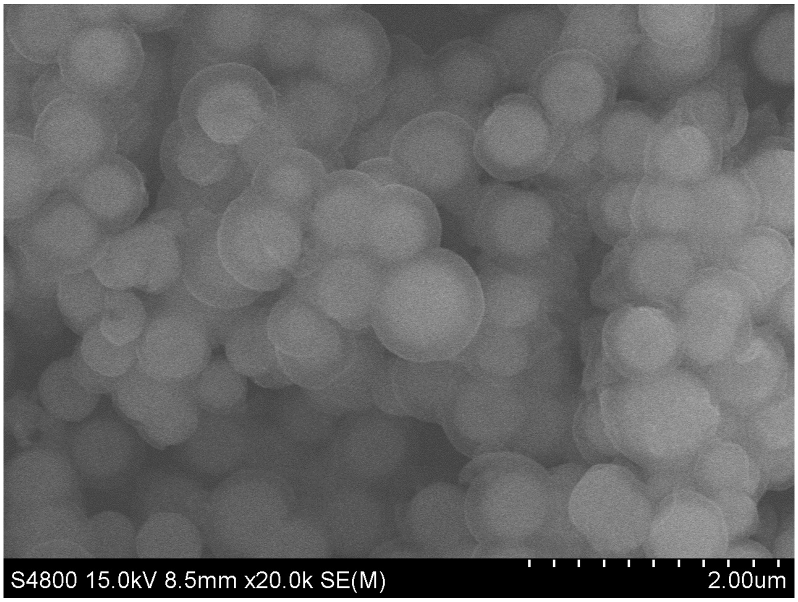

Figure S2: SEM images of the catalyst after six cycles

Table S1: EDS spectra of the catalyst after five recycles

Element	wt.%
C K	30.22
O K	25.35
Fe K	42.33
Pd L	2.10
Total	100.00

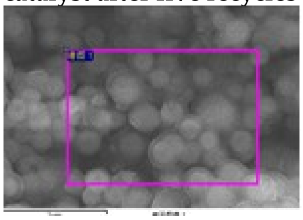

2. **Characterization of all products**

2-methyl benzaldehyde [1]: ^{1}H NMR (400 MHz, $CDCl_3$) δ 10.26 (s, 1H), 7.79 (dd, J = 7.6, 1.4 Hz, 1H), 7.47 (td, J = 7.5, 1.5 Hz, 1H), 7.36 (t, J = 7.5 Hz, 1H), 7.26 (d, J = 7.8 Hz, 1H), 2.67 (s, 3H). ^{13}C NMR (101 MHz, $CDCl_3$) δ 192.93 (s), 140.66 (s), 134.11 (s), 133.71 (s), 132.09 (s), 131.80 (s), 126.36 (s), 19.64 (s).

4-methyl benzaldehyde [1]: ^{1}H NMR (400 MHz, $CDCl_3$) δ 9.96 (s, 1H), 7.83 – 7.73 (d, J = 8.2 Hz, 2H), 7.33 (d, *J* = 8.2 Hz, 2H), 2.44 (s, 3H).^{13}C NMR (101 MHz, $CDCl_3$) δ 192.13 (s), 145.62 (s), 134.16 (s), 129.89 (s), 129.74 (s), 21.92 (s).

4-iso-propylbenzaldehyde [2]: ^{1}H NMR (400 MHz, $CDCl_3$) δ 9.97 (s, 1H), 7.86 – 7.77 (d, *J* = 7.82 Hz, 2H), 7.37 (d, *J* = 7.82 Hz, 2H), 3.03 – 2.94 (m, 1H), 1.28 (d, *J* = 6.9 Hz, 6H).^{13}C NMR (101 MHz, $CDCl_3$) δ 192.12 (s), 156.28 (s), 134.53 (s), 130.04 (s), 126.62 (s), 34.49 (s), 23.64 (s).

4-methoxybenzaldehyde [1]: ^{1}H NMR (400 MHz, $CDCl_3$) δ 9.88 (s, 1H), 7.89 – 7.78 (d, *J* = 7.84 Hz, 2H), 7.04 – 6.95 (d, *J* = 7.84 Hz, 2H), 3.89 (s, 3H).^{13}C NMR (101 MHz, $CDCl_3$) δ 190.96 (s), 164.63 (s), 132.04 (s), 129.89 (s), 114.33 (s), 55.61 (s).

4-chlorobenzaldehyde [1]: ^{1}H NMR (400 MHz, $CDCl_3$) δ 9.99 (s, 1H), 7.89 – 7.78 (d, *J* = 7.85 Hz, 2H), 7.57 – 7.48 (d, *J* = 7.84 Hz, 2H).^{13}C NMR (101 MHz, $CDCl_3$) δ 190.97 (s), 140.98 (s), 134.68 (s), 130.95 (s), 129.48 (s).

4-bromobenzaldehyde [1]: ^{1}H NMR (400 MHz, $CDCl_3$) δ 9.98 (s, 1H), 7.76 (d, *J* = 8.4 Hz, 2H), 7.69 (d, *J* = 8.5 Hz, 2H).^{13}C NMR (101 MHz, $CDCl_3$) δ 191.16 (s), 135.05 (s), 132.46 (s), 131.01 (s), 129.82 (s).

4-nitrobenzaldehyde [1]: ^{1}H NMR (400 MHz, $CDCl_3$) δ 10.18 (s, 1H), 8.41 (d, *J* = 8.7 Hz, 1H), 8.10 (d, *J* = 8.8 Hz, 1H).^{13}C NMR (101 MHz, $CDCl_3$) δ 190.40 (s), 151.11 (s), 140.04 (s), 130.53 (s), 124.34 (s).

4-biphenylcarboxaldehyde [3]: ^{1}H NMR (400 MHz, $CDCl_3$) δ 10.06 (s, 1H), 7.96 (d, *J* = 8.5 Hz, 2H), 7.75 (d, *J* = 8.2 Hz, 2H), 7.66 – 7.61 (m, 2H), 7.53 – 7.45 (m, 2H), 7.45 – 7.39 (m, 1H).^{13}C NMR (101 MHz, $CDCl_3$) δ 192.05 (s), 147.23 (s), 139.72 (s), 135.17 (s), 130.32 (s), 129.05 (s), 128.51 (s), 127.72 (s), 127.40 (s).

1-naphthaldehyde [4]: ^{1}H NMR (400 MHz, $CDCl_3$) δ 10.38 (s, 1H), 9.25 (d, *J* = 8.6 Hz, 1H), 8.08

(d, J = 8.2 Hz, 1H), 7.97 (d, J = 7.0 Hz, 1H), 7.91 (d, J = 8.2 Hz, 1H), 7.69 (ddd, J = 8.5, 6.9, 1.4 Hz, 1H), 7.65 – 7.54 (m, 2H).^{13}C NMR (101 MHz, $CDCl_3$) δ 193.68 (s), 136.81 (s), 135.37 (s), 133.73 (s), 131.39 (s), 130.53 (s), 129.13 (s), 128.52 (s), 127.01 (s), 124.91 (s).

1,4-phthalaldehyde [5]: ^{1}H NMR (400 MHz, $CDCl_3$) δ 10.15 (s, 2H), 8.07 (s, 4H).^{13}C NMR (101 MHz, $CDCl_3$) δ 191.61 (s), 139.98 (s), 130.16 (s).

2-furaldehyde [6]: ^{1}H NMR (400 MHz, $CDCl_3$) δ 9.67 (s, 1H), 7.91 – 7.57 (m, 1H), 7.28 (dd, J = 3.6, 0.6 Hz, 1H), 6.62 (dd, J = 3.6, 1.7 Hz, 1H).^{13}C NMR (101 MHz, $CDCl_3$) δ 177.93 (s), 152.94 (s), 148.14 (s), 121.20 (s), 112.63 (s).

2-thiophenecarboxaldehyde [4]: ^{1}H NMR (400 MHz, $CDCl_3$) δ 9.95 (s, 1H), 7.83 – 7.75 (m, 2H), 7.23 (dd, J = 4.9, 3.8 Hz, 1H).^{13}C NMR (101 MHz, $CDCl_3$) δ 183.11 (s), 144.01 (s), 136.46 (s), 135.21 (s), 128.38 (s).

indole-3-carboxaldehyde [7]: ^{1}H NMR (400 MHz, DMSO) δ 12.16 (s, 1H), 9.94 (s, 1H), 8.30 (s, 1H), 8.10 (d, J = 7.1 Hz, 1H), 7.52 (d, J = 7.6 Hz, 1H), 7.24 (dtd, J = 17.7, 7.2, 1.2 Hz, 2H).^{13}C NMR (101 MHz, DMSO) δ 185.44 (s), 138.99 (s), 137.50 (s), 124.56 (s), 123.93 (s), 122.60 (s), 121.29 (s), 118.61 (s), 112.89 (s).

2-pyridinecarboxaldehyde [6]: ^{1}H NMR (400 MHz, $CDCl_3$) δ 10.10 (s, 1H), 8.82 (d, J = 4.8 Hz, 1H), 7.99 (d, J = 7.8 Hz, 1H), 7.95 – 7.85 (m, 1H), 7.56 (ddd, J = 7.5, 4.8, 1.3 Hz, 1H).^{13}C NMR (101 MHz, $CDCl_3$) δ 193.43 (s), 152.73 (s), 150.20 (s), 137.14 (s), 127.94 (s), 121.75 (s).

3. NMR spectra

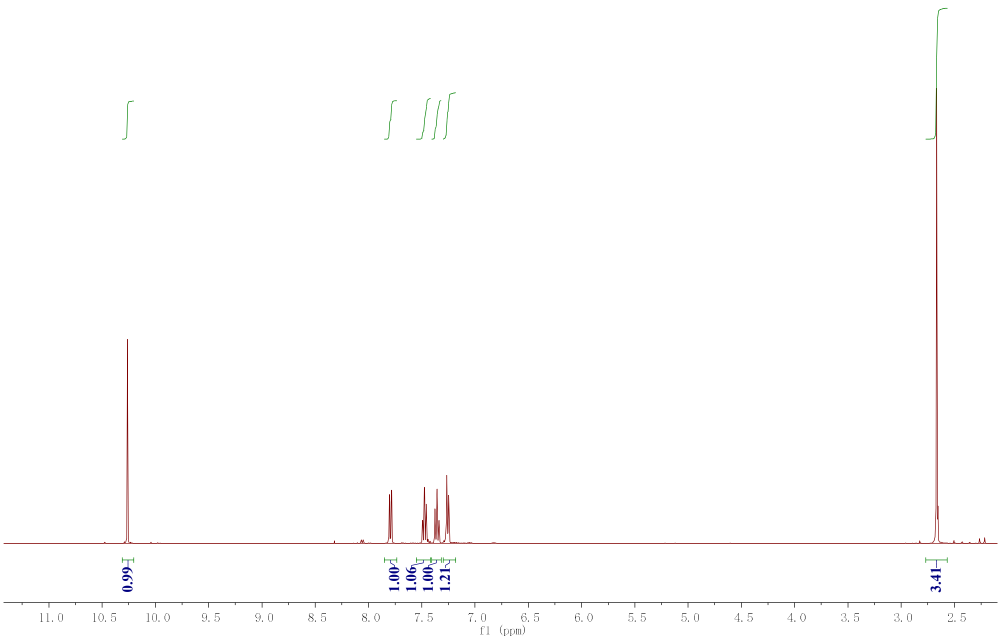

Figure S3: ^{1}H NMR **of 2-methyl benzaldehyde**

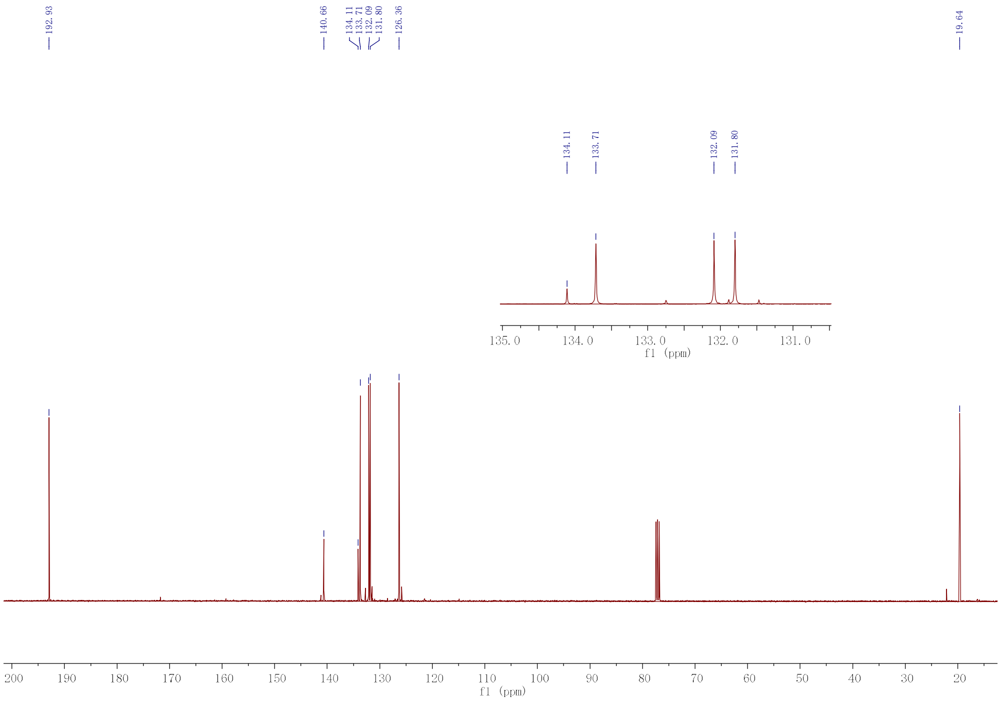

Figure S4: ^{13}C NMR **of 2-methyl benzaldehyde**

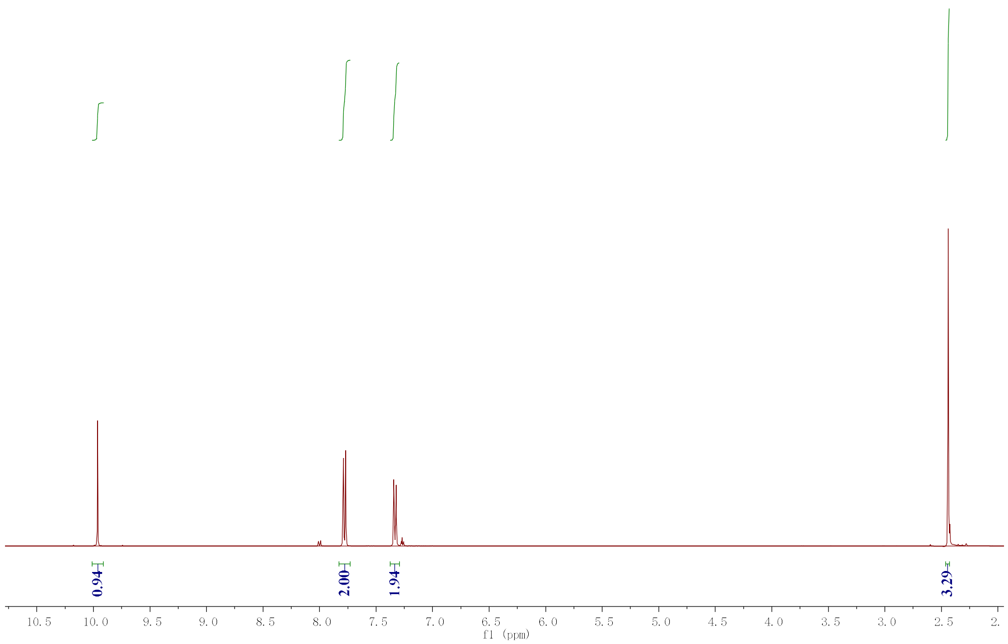

Figure S5: ¹H NMR **of 4-methyl benzaldehyde**

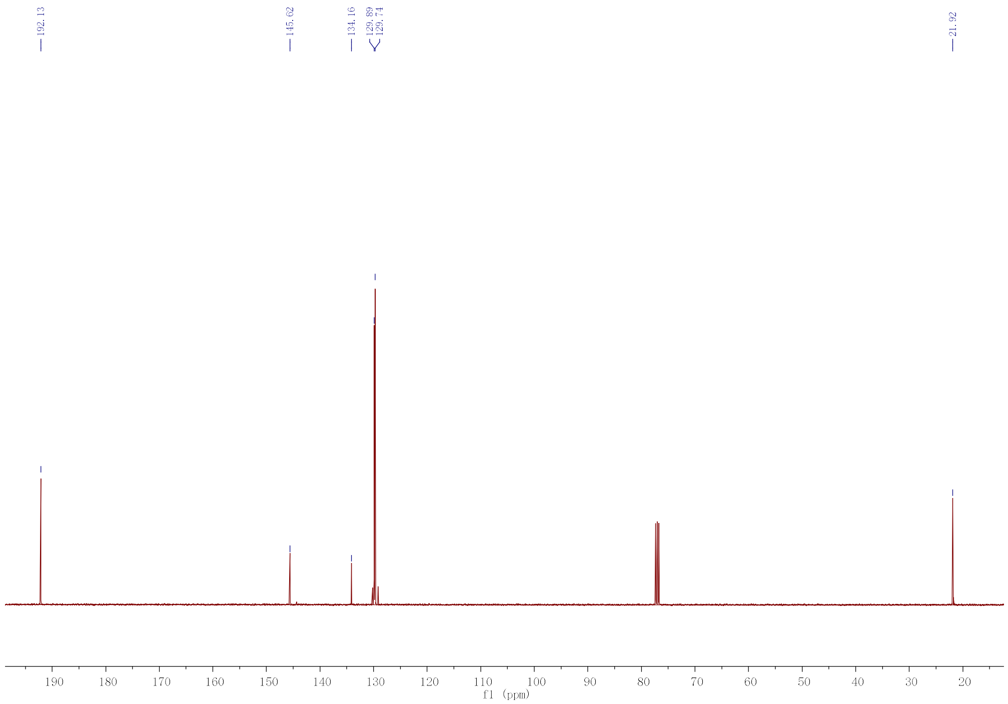

Figure S6: ¹³C NMR **of 4-methoxybenzaldehyde**

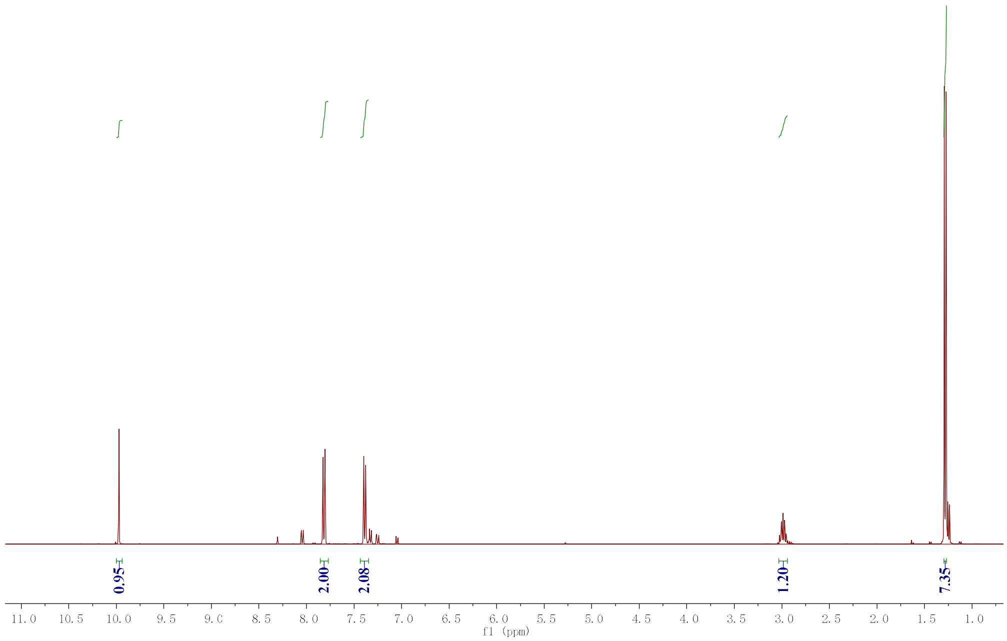

Figure S7: ^{1}H NMR **of 4-iso-propylbenzaldehyde**

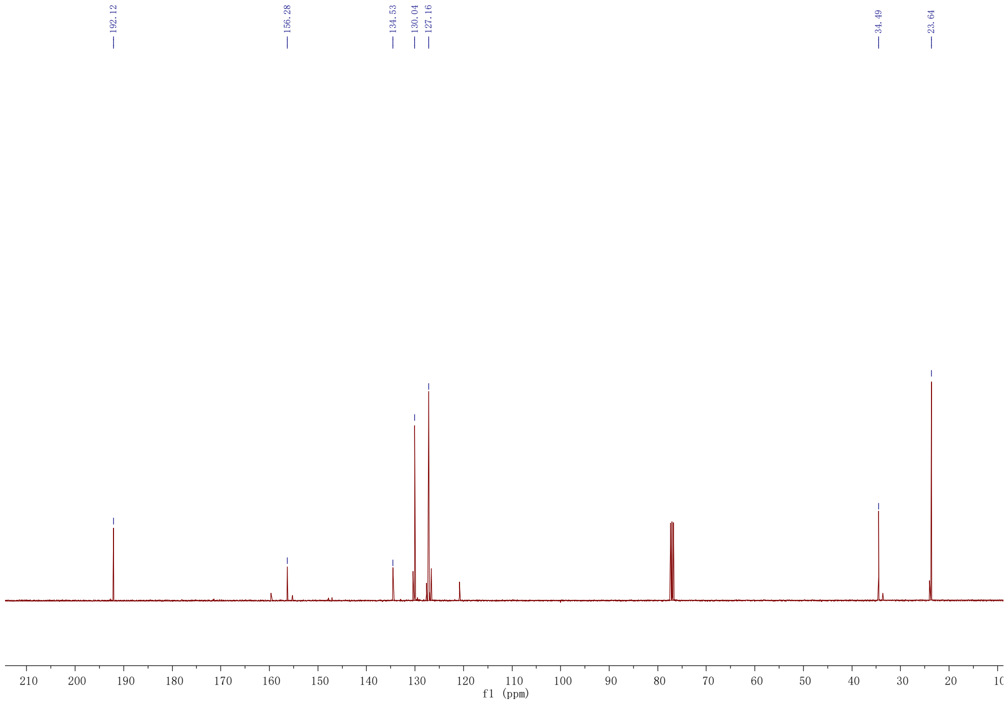

Figure S8: ^{13}C NMR **of 4-iso-propylbenzaldehyde**

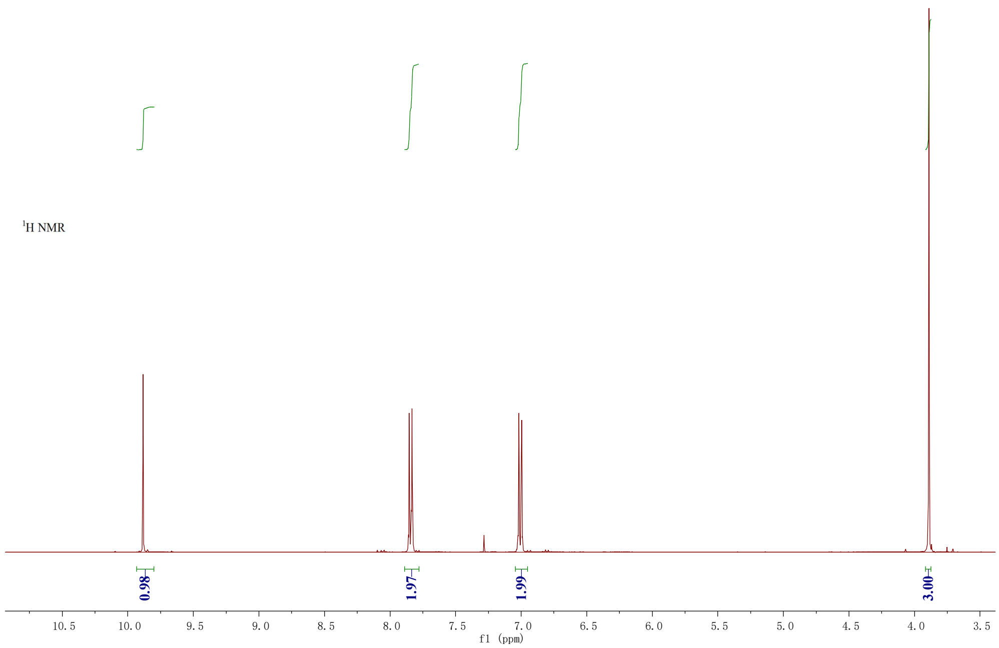

Figure S9: ^{1}H NMR **of 4-methoxybenzaldehyde**

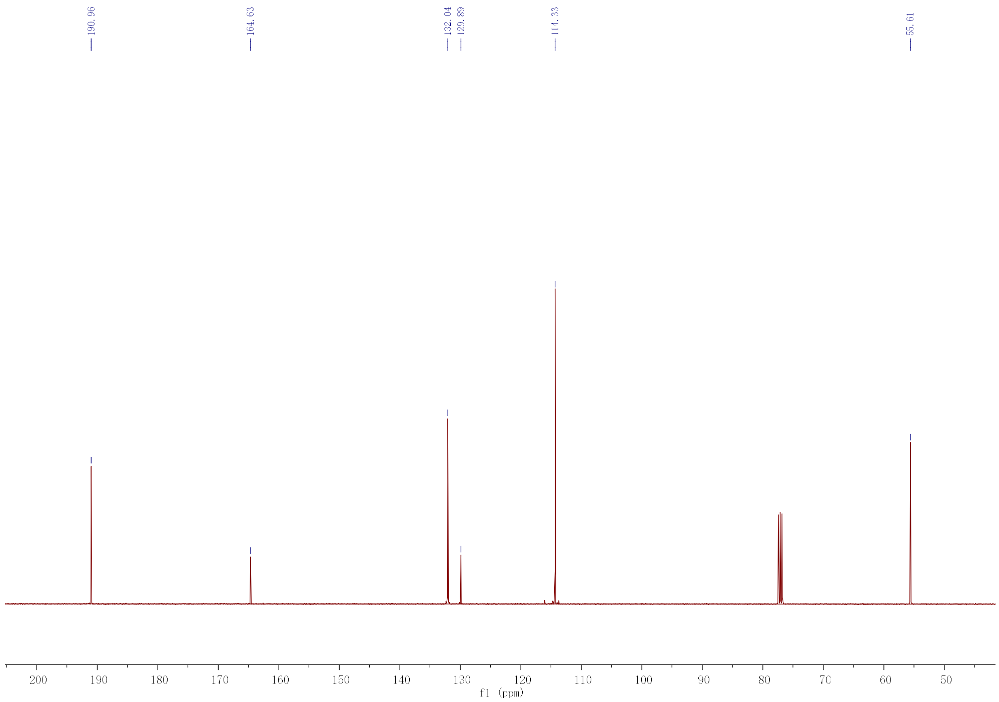

Figure S10: ^{13}C NMR **of 4-methoxybenzaldehyde**

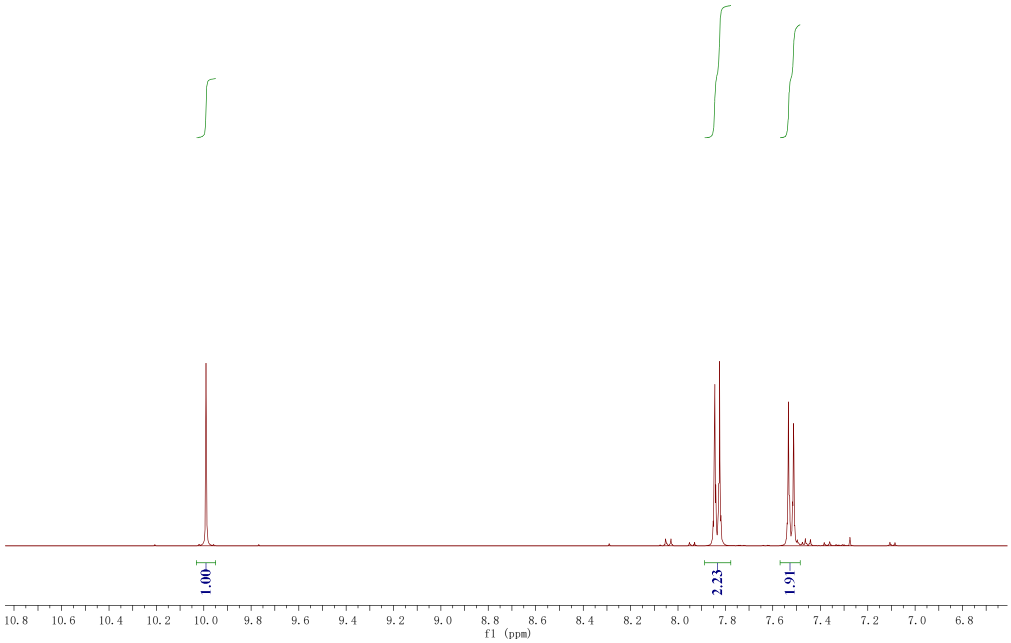

Figure S11: ^{1}H NMR **of 4-chlorobenzaldehyde**

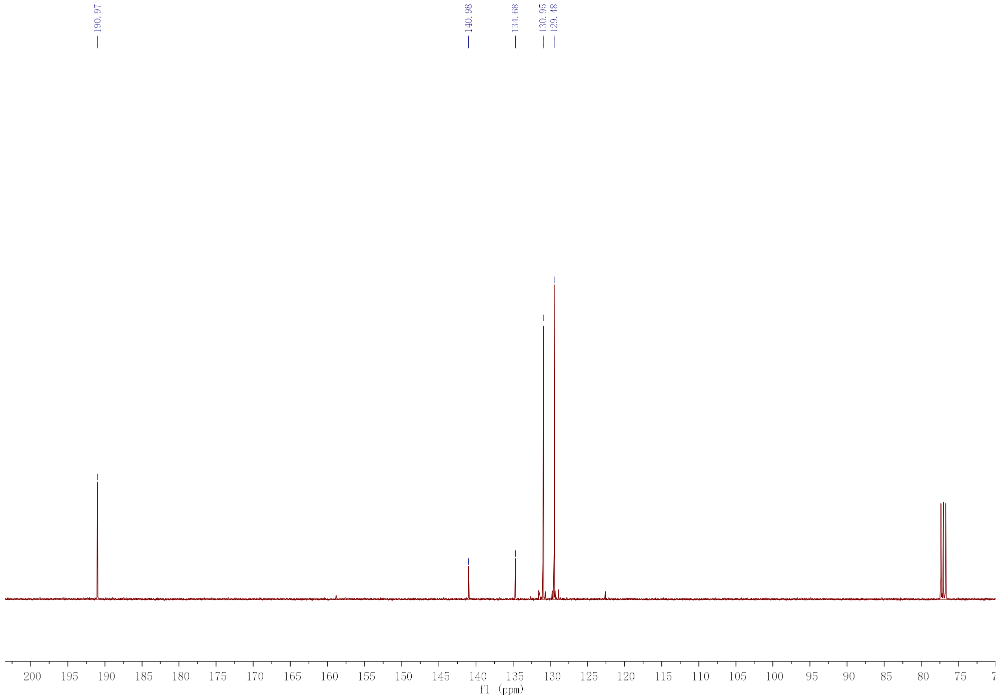

Figure S12: ^{13}C NMR **of 4-chlorobenzaldehyde**

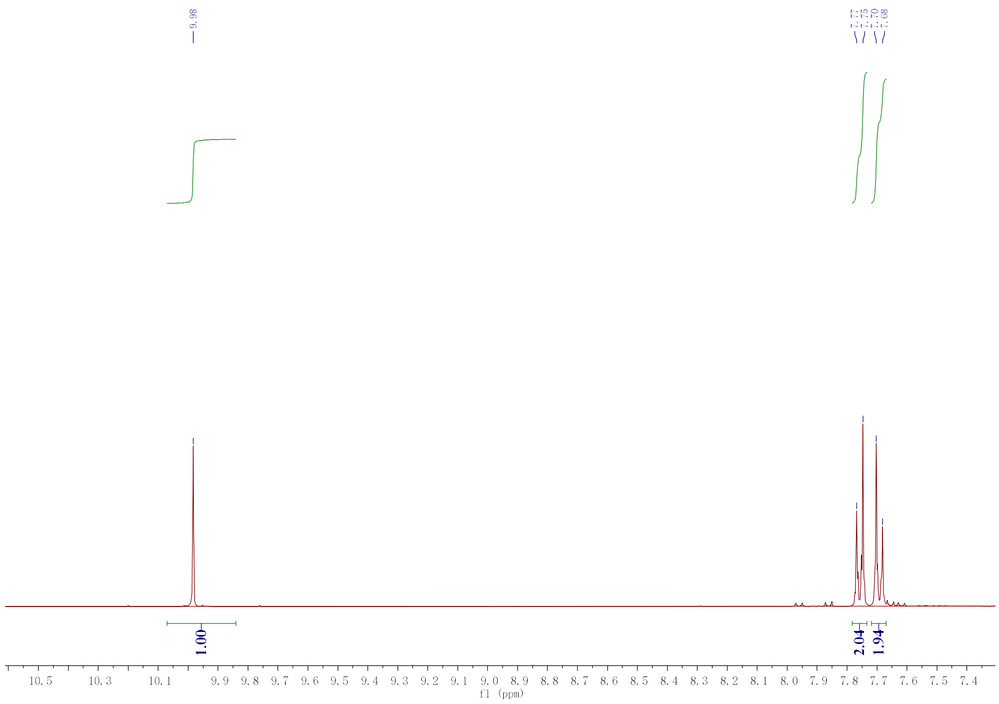

Figure S13: ^{1}H NMR **of 4-bromobenzaldehyde**

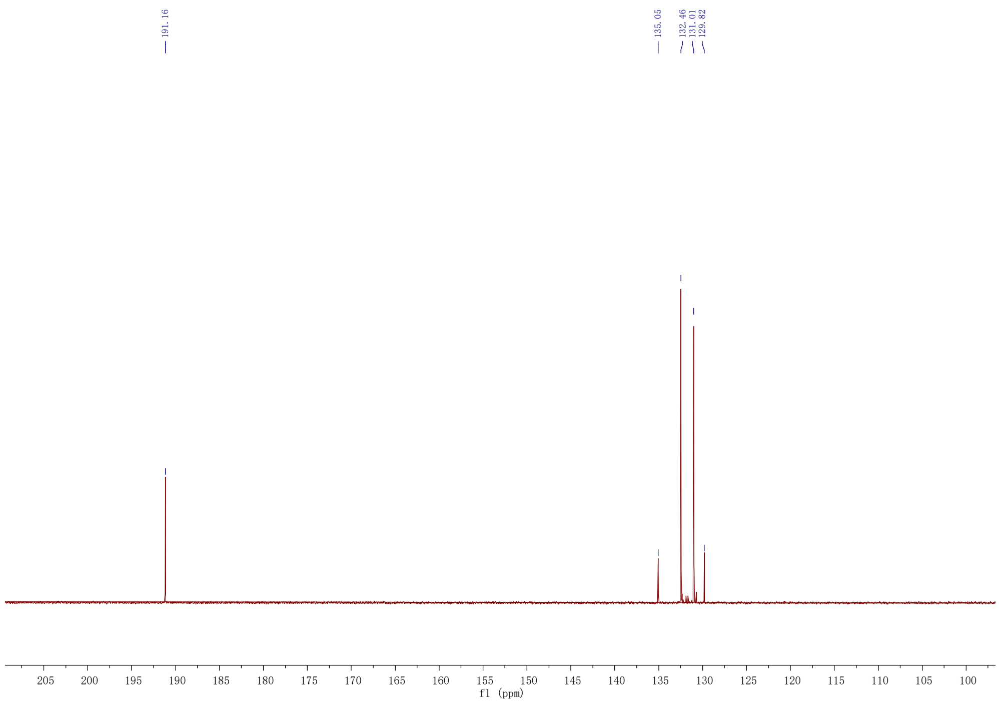

Figure S14: ^{13}C NMR **of 4-bromobenzaldehyde**

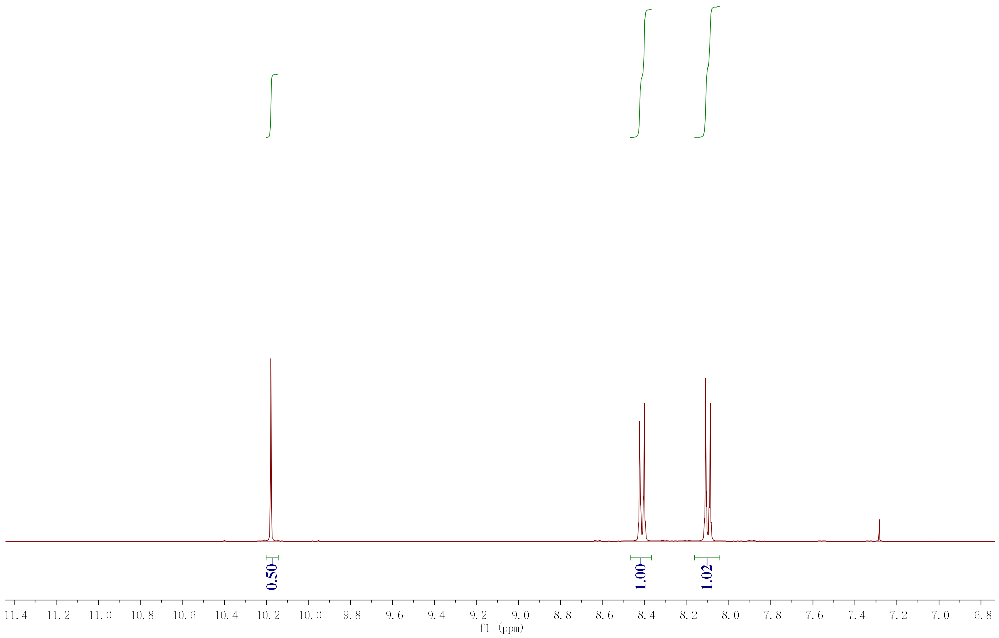

Figure S15: ^{1}H NMR **of 4-nitrobenzaldehyde**

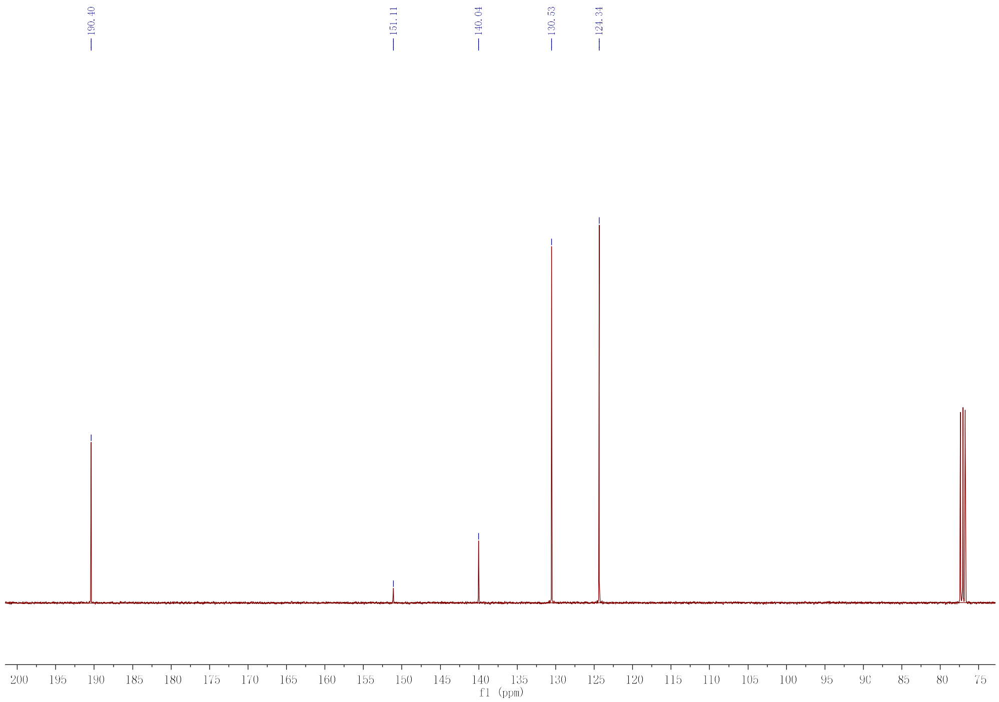

Figure S16: ^{13}C NMR **of 4-nitrobenzaldehyde**

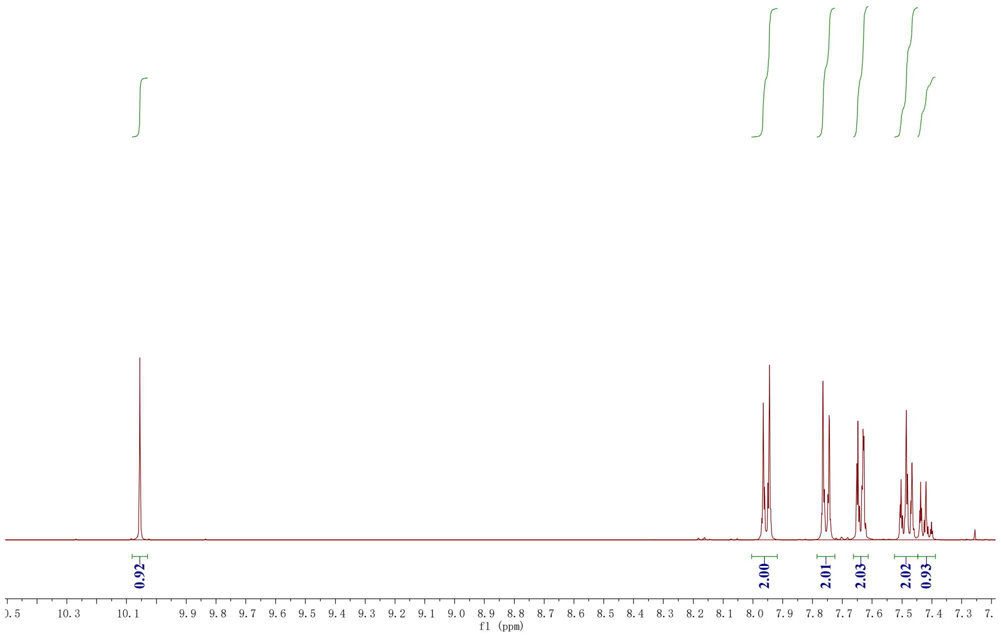

Figure S17: ^{1}H NMR **of 4-biphenylcarboxaldehyde**

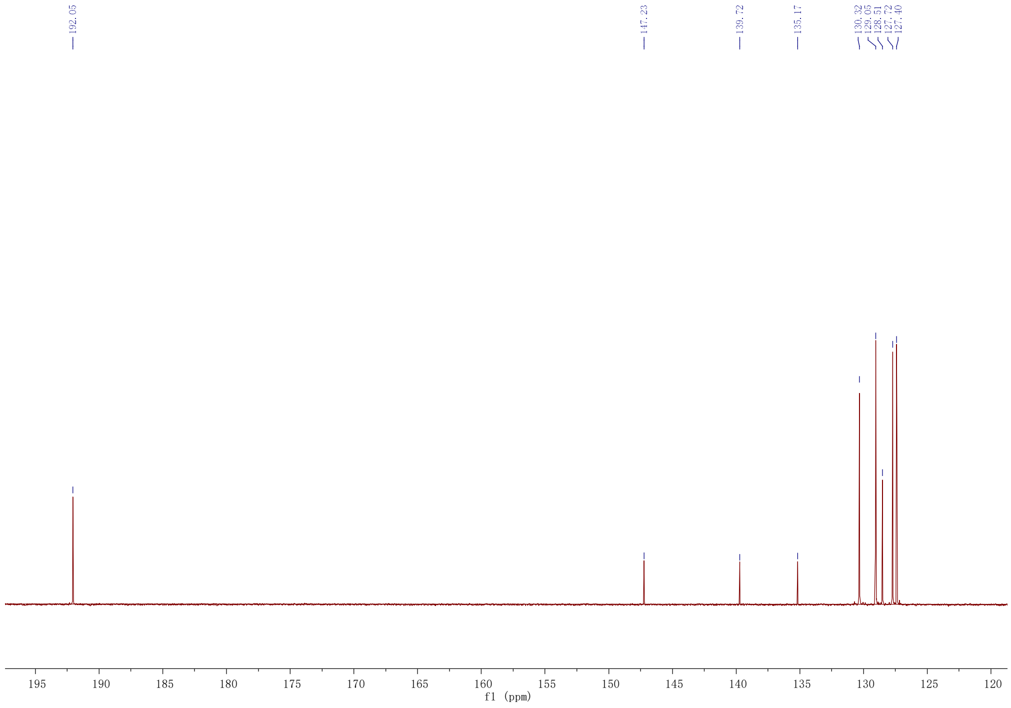

Figure S18: ^{13}C NMR **of 4-biphenylcarboxaldehyde**

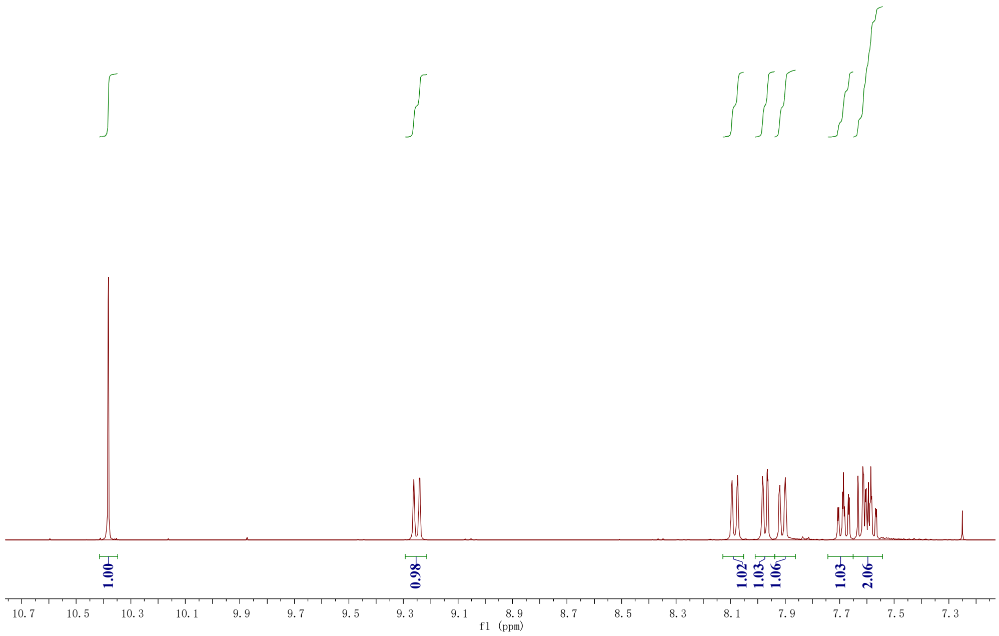

Figure S19: ^{1}H NMR **of 1-naphthaldehyde**

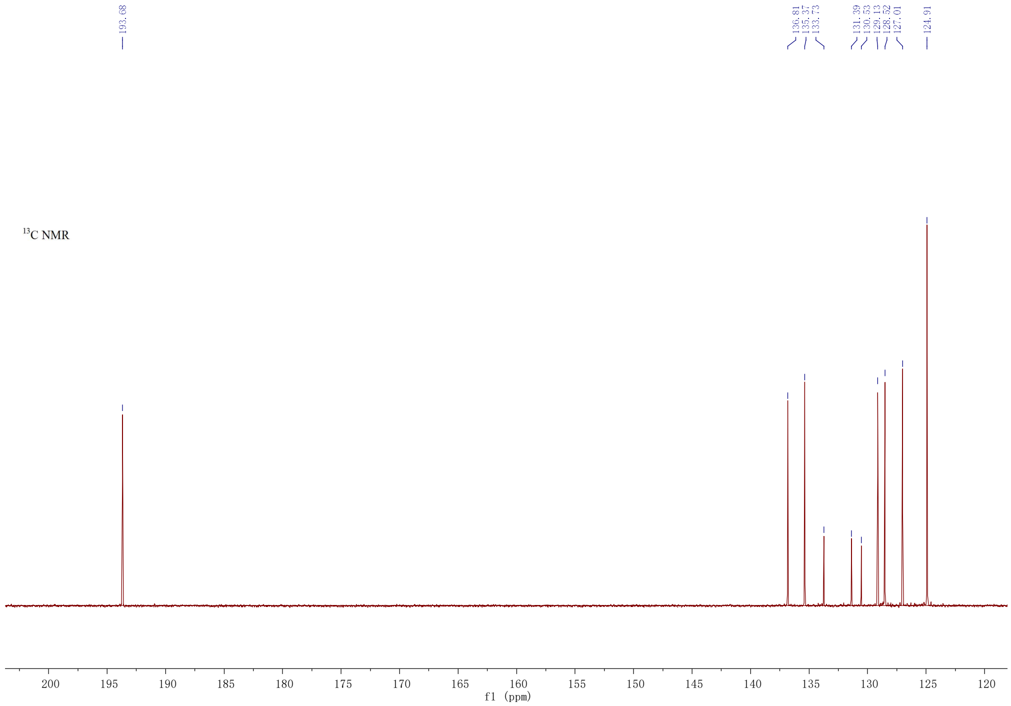

Figure S20: ^{13}C NMR **of 1-naphthaldehyde**

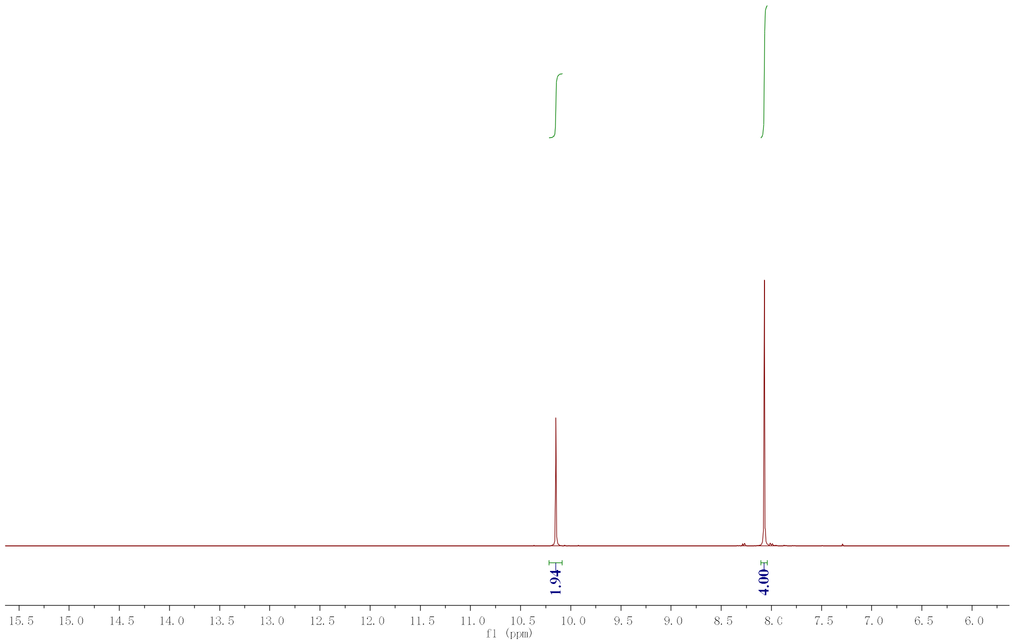

Figure S21: ^{1}H NMR **of 1,4-phthalaldehyde**

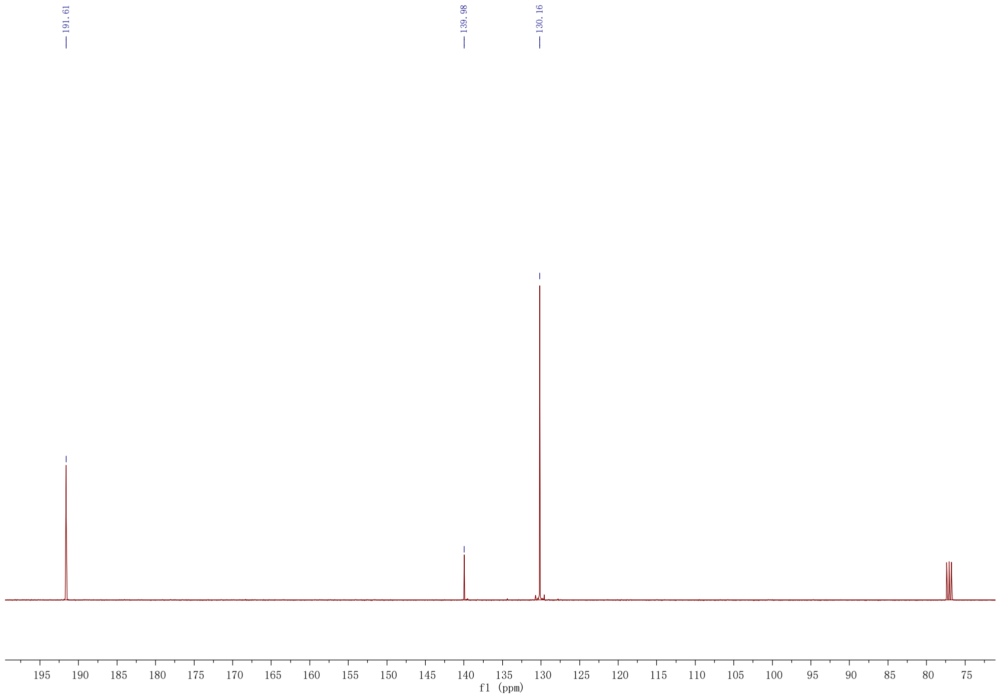

Figure S22: ^{13}C NMR **of 1,4-phthalaldehyde**

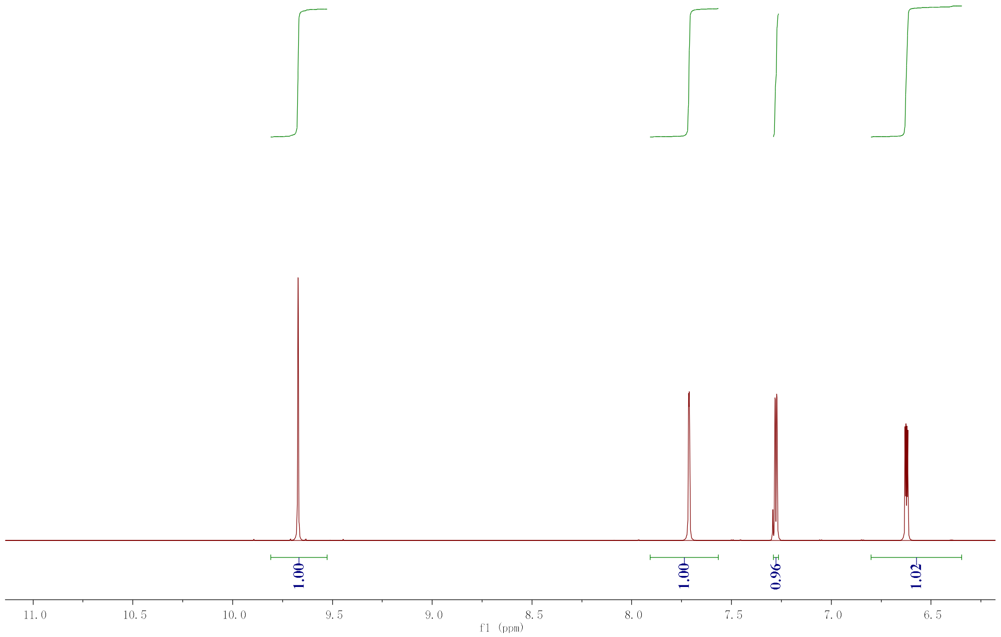

Figure S23: ^{1}H NMR **of 2-furaldehyde**

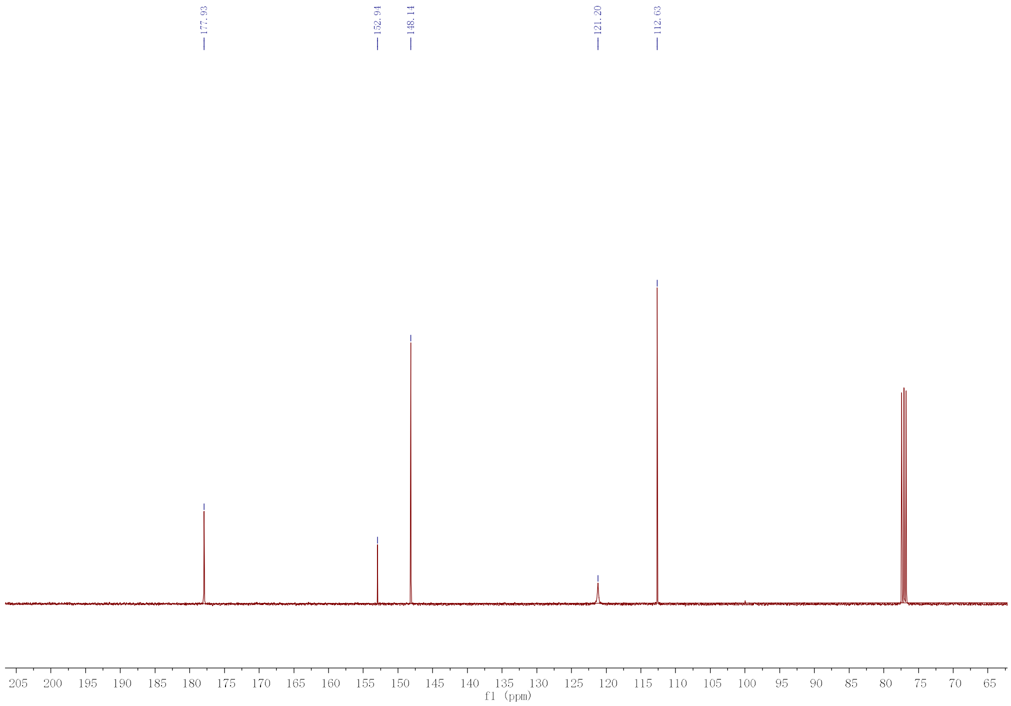

Figure S24: ^{13}C NMR **of 2-furaldehyde**

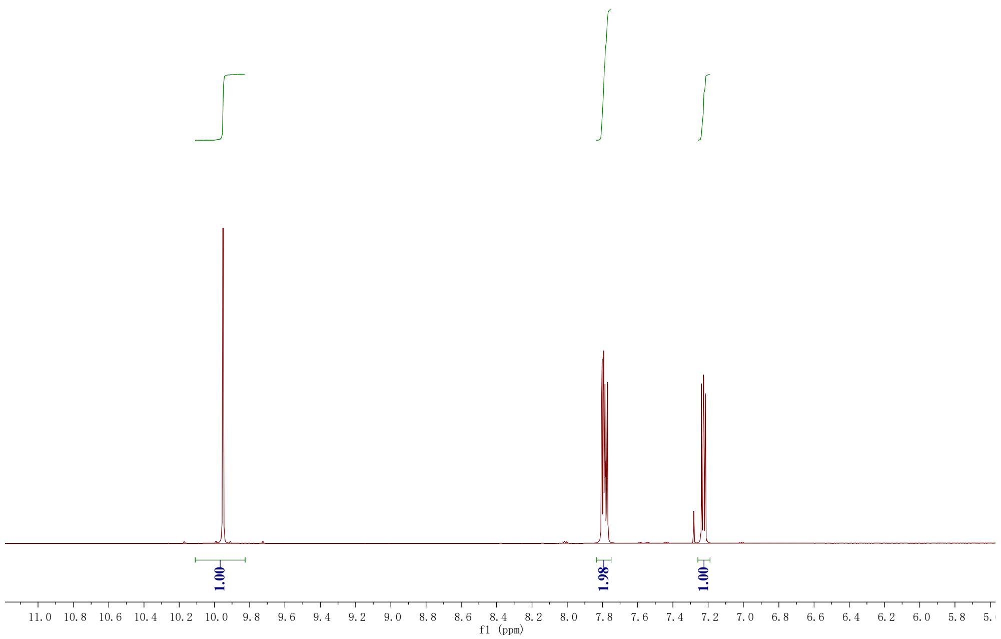

Figure S25: ¹H NMR **of 2-thiophenecarboxaldehyde**

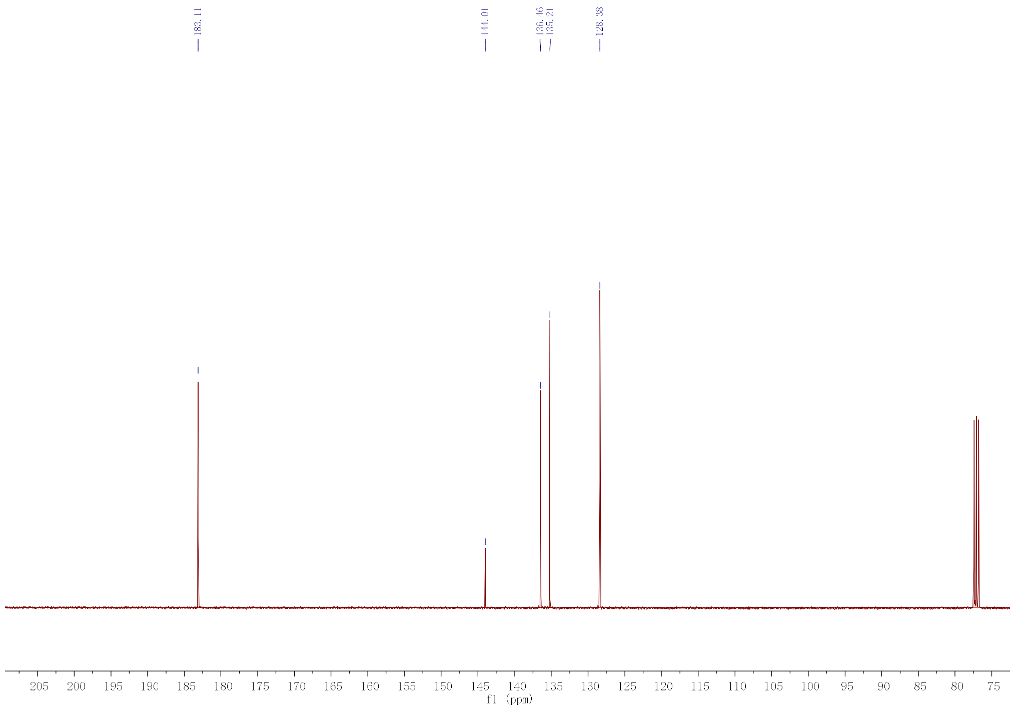

Figure S26: ¹³C NMR **of 2-thiophenecarboxaldehyde**

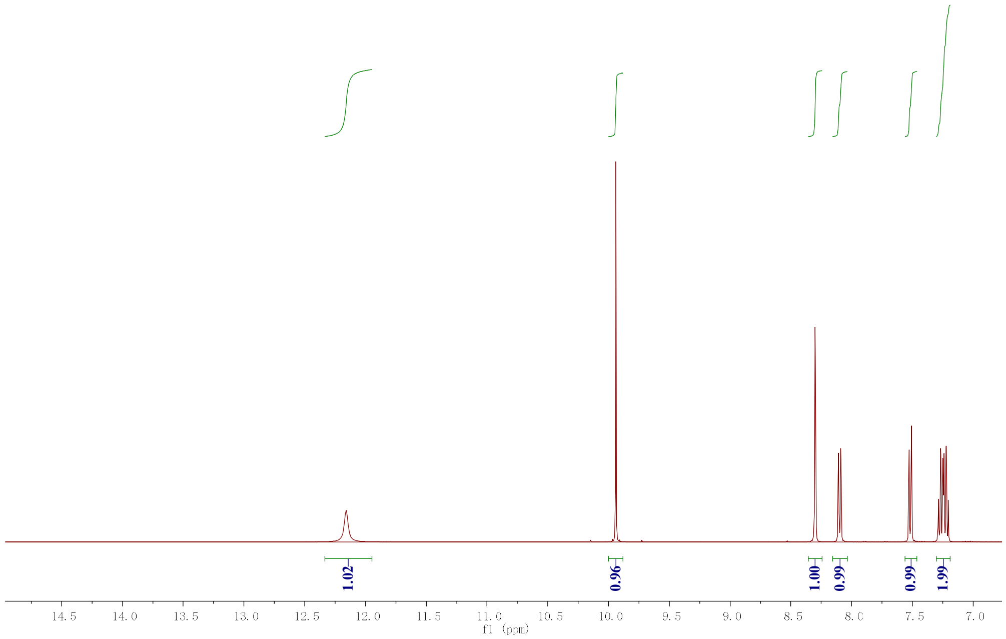

Figure S27: ^{1}H NMR **of indole-3-carboxaldehyde**

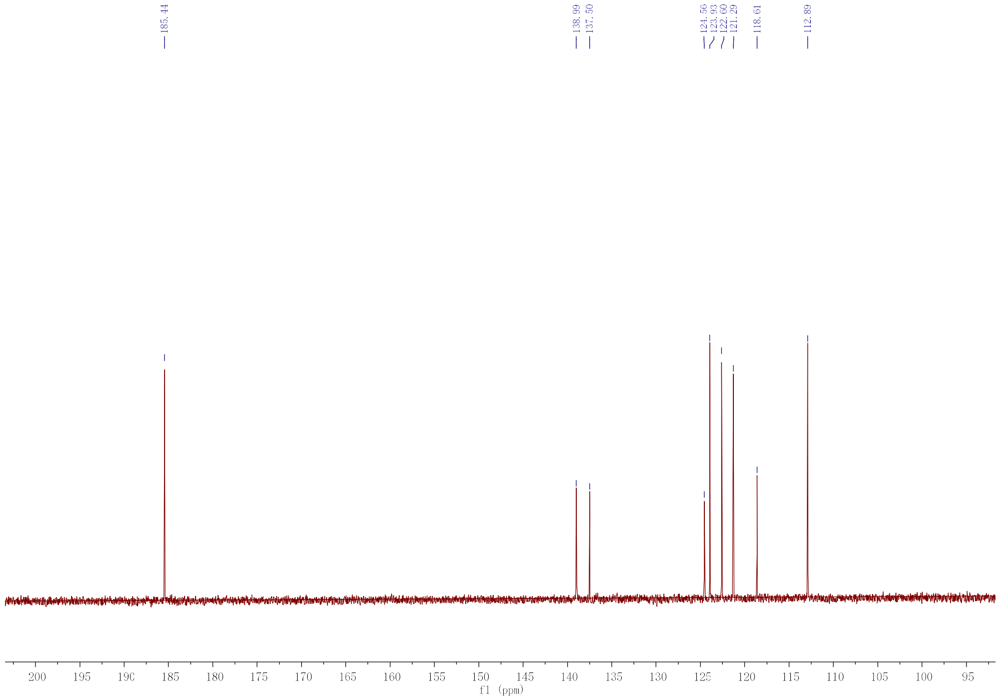

Figure S28: ^{13}C NMR **of indole-3-carboxaldehyde**

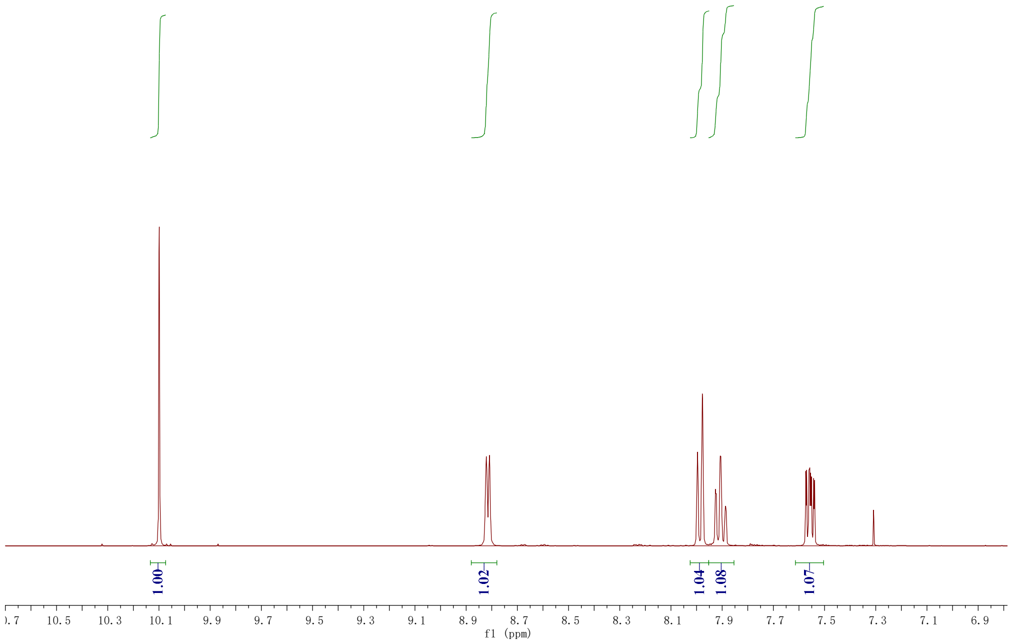

Figure S29: ^{1}H NMR **of 2-pyridinecarboxaldehyde**

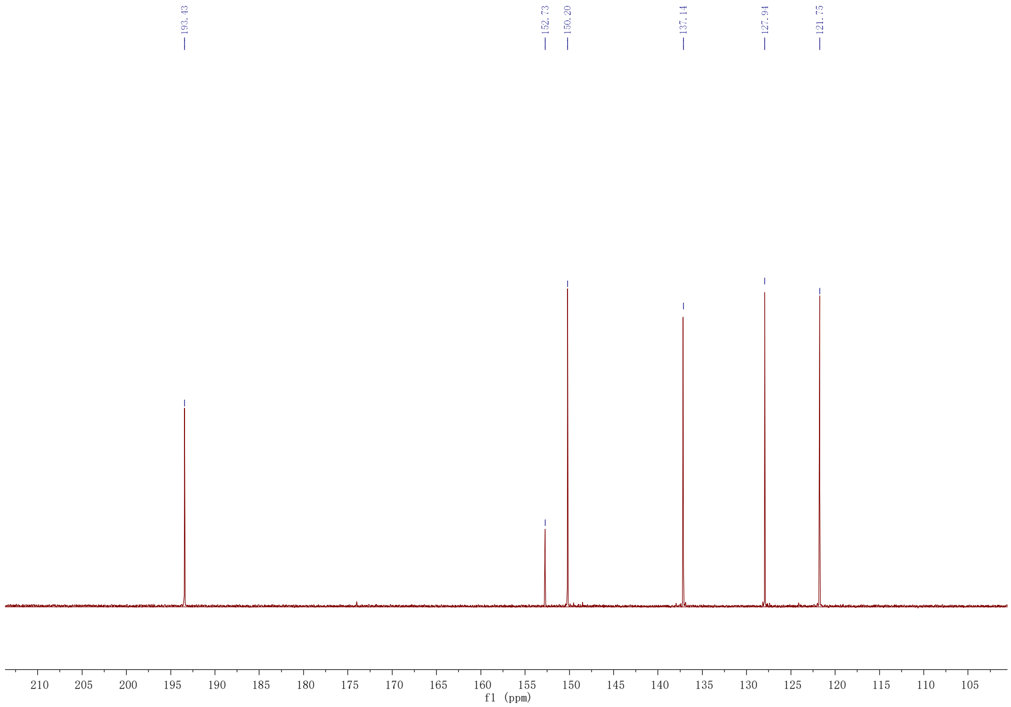

Figure S30: ^{13}C NMR of 2-pyridinecarboxaldehyde

4. References

[1] Baillargeon, V.P.; Stille, J.K. Palladium-catalyzed formylation of organic halides with carbon monoxide and tin hydride. *J. Am. Chem. Soc.* **1986**, *108*, 452-461.

[2] Han, C.; Buchwald, S.L. Negishi coupling of secondary alkylzinc halides with aryl bromides and chlorides. *J. Am. Chem. Soc.* **2009**, *131*, 7532-7533.

[3] Hong, B.C.; Tseng, H.C.; Chen, S.H. Synthesis of Aromatic Aldehydes by Organocatalytic [4+2] and [3+3] cycloaddition of α,β-unsaturated aldehydes. *Tetrahedron* **2007**, *63*, 2840–2850.

[4] Kyoungsoo, L.; Maleczka, R.E. Pd(0)-Catalyzed PMHS reductions of aromatic acid chlorides to aldehydes. *Org. Lett.* **2006**, *8*, 1887-1888.

[5] Pelletier, G.; Bechara, W.S.; Charette, A.B. Controlled and chemoselective reduction of secondary amides. *J. Am. Chem. Soc.* **2010**, *132*, 12817-12819.

[6] Velusamy, S.; Ahamed, M.; Punniyamurthy, T. Novel polyaniline-supported molybdenum-catalyzed aerobic oxidation of alcohols to aldehydes and ketones. *Org. Lett.* **2004**, *6*, 4821-4824.

[7] Duarte, N.; Ferreira, M.J. Lagaspholones A and B: two new jatropholane-type diterpenes from euphorbia lagascae. *Org. Lett.* **2007**, *9*, 489-492.
